# Psychometric properties of the Turkish version of the Anxiety and Preoccupation about Sleep Questionnaire in clinical and non-clinical samples

**DOI:** 10.5935/1984-0063.20210033

**Published:** 2022

**Authors:** Ömer Faruk Uygur, Fatma Özlem Orhan, Hilal Uygur, Ali Kandeger, Onur Hursitoglu

**Affiliations:** 1 Dr. Ersin Arslan Training and Research Hospital, Gaziantep, Turkey, Department of Psychiatry - Gaziantep - Sahinbey - Turkey.; 2 Faculty of Medicine, Kahramanmaras Sütçü Imam University, Kahramanmaras, Turkey, Department of Psychiatry - Kahramanmaras - Avsar Kampüsü - Turkey.; 3 Faculty of Medicine, Selçuk University, Konya, Turkey, Department of Psychiatry - Konya - Selçuklu - Turkey.; 4 Kahramanmaras Necip Fazil City Hospital, Kahramanmaras, Turkey, Department of Psychiatry - Kahramanmaras - Dulkadiroglu - Turkey.

**Keywords:** Insomnia, anxiety, worry, factor analysis, Anxiety and Preoccupation about Sleep Questionnaire, reliability

## Abstract

**Objectives:**

The aim of the study was to investigate the psychometric properties of the Turkish version of Anxiety and preoccupation about sleep questionnaire (APSQ) in clinical and non-clinical samples.

**Material and Methods:**

Two samples (141 university students and 42 patients with major depressive disorders) completed Turkish APSQ, the Pittsburgh sleep quality index (PSQI), the insomnia severity index (ISI) and the sociodemographic data form. Content validity analysis was performed with the Davis technique after the translation process of the original scale. Explanatory factor analysis and principal component analysis were performed to determine the scales construct validity, and internal consistency and temporal stability analyses were conducted to evaluate its reliability. The PSQI and the insomnia severity index (ISI) were used to assess criterion- related validity. In addition, we divided all the participants into two groups as good-sleepers and clinical insomnia according to ISI scores. Predictive validity analyses were also computed via comparing groups.

**Results:**

Confirmatory factor analysis showed that the scale model aligns well with the original scales 10 items and two-factor structure. The scales and subdimensions Cronbach’s alpha coefficients were excellent (Factor 1; 0.935, factor 2; 0.906, total scale; 0.952). The test-retest correlations were 0.661 and 0.828 for depression group and university student group, respectively. Turkish APSQ scores were found to be significantly higher in both of the clinical groups (depression group vs. university student group, clinic insomnia group vs. good-sleepers group).

**Conclusion:**

The Turkish APSQ is adequate reliability and validity for assessing anxiety and preoccupation about sleep in Turkish clinical and non-clinical samples.

## INTRODUCTION

Insomnia is a condition characterized by difficulty falling asleep or maintaining sleep or early-morning awakening with an inability to return to sleep. When such symptoms occur at least three times a week for more than three months, they meet the diagnostic criteria of insomnia disorder, which has an estimated prevalence of 10% according to large multicenter studies^1,2^. Insomnia is associated with many health problems, including impaired daytime functioning, mood disturbance, reduced cognitive functions, altered immune function, and fatigue, and has many direct and indirect negative financial consequences. Insomnia disorder is therefore considered one of the most serious public health problems^3,4^.

Many theories aim to explain the development and maintenance of insomnia. Among these, the cognitive model - which purports that worry, selective attention, and the monitoring of these cognitive processes are critical in the persistence of insomnia - has been attracting attention in recent years^5^. According to this model, worry is the starting point of a vicious circle, as it activates the sympathetic nervous system, causing increased arousal and emotional distress. Then, selective attention on insomnia increases, which (along with arousal) leads to heightened awareness of factors that disrupt sleep, such as bodily sensations or sounds, causing more worry. Selective attention can also create bias or misperceptions about insomnia or its consequences, increasing worry. The cognitive model of insomnia thus argues that the combination of worry, arousal, and emotional stress increases anxiety, causing difficulty achieving or maintaining sleep^6-8^. In addition, this model suggests that anxiety about insomnia encourages sleep-related safety behaviors, which are adopted to prevent worry about insomnia but are not demonstrably beneficial to sleep. For example, drinking alcohol is a classic sleep-related safety behavior, but though it may initially facilitate sleep, alcohol disrupts sleep structure, causing more nighttime awakenings. Such safety behaviors do not improve sleep and can lead to additional mental health problems such as substance abuse^9,10^.

Given the importance of the relationship between insomnia and worry, there is a need to develop and analyze psychometric tools that assess insomnia worry for research and clinical purposes. Pre-sleep arousal scale (PSAS), thought control questionnaire - insomnia revised (TCQI-R), insomnia daytime worry scale (IDWS) are scales developed and validated for insomnia-related worry. The PSAS, TCQI-R, and IDWS have some limitations. The PSAS has a cognitive subscale, but it focuses on pre-sleep worry and does not measure daytime worry. The TCQI-R’s worry subscale evaluates worry management but not the presence and intensity of sleep- related worry, while the IDWS focuses only on daytime worry, not on nighttime worry^11-13^.

A fourth insomnia worry scale, the anxiety and preoccupation about sleep questionnaire (APSQ), was created by Tang and Harvey (2004)^14^ to assess worry about sleep. It measures two subdimensions - worries about the consequences of poor sleep and worries about the uncontrollability of sleep. The scale’s validity and reliability have been evaluated in a large sample, and it has been shown to correlate with anxiety and sleep quality^15^.

The APSQ is promising compared to other insomnia-related worry scales, as it is short and designed to measure both the intensity of anxiety and preoccupation with insomnia. Most importantly, it addresses both daytime and nighttime sleep-related anxiety^16^. Additionally, it is sensitive to the effects of treatment. These features make the APSQ potentially useful for research and clinical purposes^15,16^. Also, the APSQ-brief version (2-item form) displayed acceptable psychometric properties^17^.

The items of any sleep scales can mean different in various languages and the scale suitable for one society may not be suitable for the other one, and therefore some items need to be modified. Also determining psychometric properties is very important to understand how much the scale can measure the subject to be assed, whether it is reliable or not, whether it can be applied again after a certain period. From this point of view, we aimed to first perform the Turkish translation process of the original APSQ and then investigate the psychometric properties of the 10-item Turkish version of the APSQ in clinical (among patients with the major depressive disorder) and non-clinical samples (among university students).

## MATERIAL AND METHODS

### Participants and study protocol

The study sample included 183 clinical and non-clinical participants. Of these, 42 were patients diagnosed with major depression based on the fifth revision of the diagnostic and statistical manual of mental disorders (DSM-5) and admitted to the psychiatric clinic of Kahramanmaraş Necip Fazil City Hospital. The remaining 141 were non-clinical volunteers recruited from various faculties of Kahramanmaraş Sütcü Imam University. There were no specific inclusion or exclusion criteria for non-clinical participants. Inclusion criteria for the clinical group were an age between 18 and 65 and a diagnosis of major depressive disorder (MDD) according to the DSM-5^1^. Clinical and non-clinical participants were informed about the study, their written consent was obtained and then they completed a psychological test battery including, the socio-demographic form, Turkish APSQ, insomnia severity index (ISI), Pittsburg sleep quality index (PSQI). Students filled the psychological test battery in a quiet classroom environment, and the patients filled it in the outpatient clinic. To examine test-retest reliability, the Turkish APSQ was readministered to 20 randomly selected MDD patients and 25 randomly selected healthy volunteers fifteen days should be written instead of three weeks. after the initial survey.

The study was conducted between June 2019 to December 2019, and the study protocol was approved by the local ethics committee of Kahramanmaras Sutcu Imam University (approval number: 29.05.2019/10; decision number: 05).

### Translation process and content validity

Permission to adapt the APSQ to Turkish was obtained via email from Allison G. Harvey (2002)^5^, who developed the original scale. The scale was independently translated from English into Turkish by three Turkish native speakers. These translations were examined by the research team, who formed the Turkish scale by adopting the Turkish expressions determined to best represent each English item. The Turkish form of the scale was then translated back into English by a native English speaker, and the original scale was compared with this translation for consistency. Finally, the scale items were discussed by the research team and final versions were chosen.

After this translation process, the Davis technique was used to assess the scale’s content validity. Through this technique, which has experts evaluate a scale’s suitability and understandability, the scale’s 10 items were examined by 10 specialist psychiatrists who determined whether the content of items was appropriate, highly appropriate, slightly appropriate, in need of serious review, or non-compliant^18^. Based on these ratings, a content validity index was calculated. Next, a pilot study that included face-to-face interviews with 10 university students and 10 MDD patients assessed how subjects understood each item.

### Measurement tools

#### Anxiety and preoccupation about sleep questionnaire

The 10-item APSQ was developed by Tang and Harvey (2004)^14^ to measure sleep-related anxiety. Participants rate how true each statement is for them during the last month on a 10-point Likert-type scale (from 1 “strongly disagree” to 10 “strongly agree”). The scale has two subdimensions. Items 1-5 and 7 measure the first subdimension (worries about the consequences of poor sleep), while items 6 and 8-10 measure the second (worries about the uncontrollability of sleep). Summing the ratings of all items yields a total scale score. Higher APSQ scores indicate higher insomnia-related anxiety and preoccupation. Sample items are “I worry about the amount of sleep I am going to get every night” and “I worry about my loss of control over sleep”. The internal consistency of the scale is 0.92, and its psychometric properties have been examined in a large sample. The APSQ has been found to correlate with cognitive arousal, sleep quality, sleep-related beliefs, anxiety, and depression^15^.

#### Insomnia severity index

The ISI is a measurement tool with highly valid and reliable that evaluates the severity of insomnia. The index consists of 7 questions and each is scored between 0 and 4 points. The total score ranges from 0 to 28 points. The higher the total score, the greater the severity of insomnia. Scores of 15 and above are considered as clinical insomnia^19^. Boysan et al. (2010)^20^ conducted the Turkish version of the index.

#### Pittsburg sleep quality index

The PSQI is a self-administered questionnaire that evaluates sleep quality and disturbances over a one-month time interval^21^. The scale consists of twenty-four items, nineteen of which are self-assessment questions also five of which are questions answered by a roommate or a subject’s spouse. Self-rated nineteen questions include the following seven subscales: (i) positive quality of sleep; (ii) sleep latency; (iii) sleep duration; (iv) sleep efficiency; (v) sleep disturbances; (vi) medication use for sleep; and (vii) daytime dysfunction. The seven subscales are scored between 0 and 3 points. The total score ranges from 0 to 21 points with high scores indicate poor sleep quality. The validity and reliability of the PSQI was shown acceptable in Turkish population^22^.

### Statistical analysis

The Statistical Package for the Social Sciences (SPSS) version 23 and SPSS Amos 24 were used to analyze the data from the present study. The sample variances were first evaluated for normality and homogeneity, and all were in acceptable ranges. The presence of outliers was also controlled. To meet the normal distribution assumption, kurtosis and skewness parameters were set within ±2^23^.

We started by evaluating participants’ sociodemographic characteristics using descriptive statistics (number, percentage, average, and standard deviation). We used the Kaiser-Meyer-Olkin (KMO) test to assess the adequacy of the sample size^24^. Suitability of the scale for factor analysis was evaluated via Bartlett’s test, and explanatory factor analysis (EFA) was first applied to determine the factor structure^25^. Principal component analysis (PCA) and direct oblimin rotation methods were used in the EFA. As confirmatory factor analysis (CFA) is recommended to further support models, CFA was performed via SPSS Amos 24 to test the factor structure obtained via EFA Many fit indices are used in CFA to reveal the adequacy and compatibility of the tested model^26,27^.

Our analyses included the chi-square fit test, the comparative fit index (CFI), the general fit index (GFI), the root mean square error of approximation (RMSEA), and the incremental fit index (IFI). Cronbach’s alpha and item-total score correlations were used to determine the scale’s reliability, and its temporal stability was assessed via the test-retest method. Fifteen days after the baseline assessment, the Turkish APSQ was readministered to 20 randomly selected MDD patients and 25 randomly selected controls.

To determine the Turkish APSQ’s criterion validity, we examined its relationship to scores on the PSQI, and the ISI using Pearson correlations. An independent samples t-test was computed to compare the APSQ scores of MDD patients and controls to assess the scale’s predictive validity. We divided all participants into good-sleepers group and clinical insomnia group according to ISI scores that 15 point and above indicates clinical insomnia. Then we compared mean scores of sleep measures between these groups via independent samples t-test.

## RESULTS

### Description statistics and sleep parameters

All participants’ ages ranged from 18 to 53 with a mean of 24.10 years (SD ± 8.30). Most were female (*n*=140, 76.5%) and university students (*n*=141, 82%). The mean age of the control group was 20.5, while the mean age in the depression group was 35.9. Most of the control group and depression group were women. Sleep parameters of the depression group were worse than the control group. Also, the clinical insomnia rate was higher in the depression group according to the ISI. Table 1 shows the sociodemographic data and sleep parameters of both groups.

**Table 1:** Sociodemographic data and sleep parameters.

	Control group (n=141)	Depression group (n=42)
Age (M (SD))	20.5 (1.84)	35.9 (10.3)
Gender (female)	%78	%71.4
Clinical insomnia	%17.8	%75
PSQI M(SD)	5.90 (4.58)	13.69 (3.43)
ISI M(SD)	9.52 (5.80)	17.78 (4.96)
APSQTOTAL M(SD)	33.35 (24.92)	67.54 (24.61)
APSQF1 M(SD)	21.47 (16.14)	37.78 (16.62)
APSQF2 M(SD)	11.41 (10.04)	25.83 (11.10)

**Notes:** APSQ = Anxiety and preoccupation about sleep questionnaire; F1 = Factor 1: worries about the consequences of poor sleep; F2 = Factor 2: worries about uncontrollability of sleep; PSQI = Pittsburg sleep quality index; ISI = Insomnia severity index; n = Number of individuals; M = Mean; SD = Standard deviation.

### Content validity

Based on expert scores, each item on the Turkish APSQ yielded a content validity index above 0.8, exceeding accepted standards^18^. Thus, no item was removed. From a pilot administration with 20 individuals, it was determined that all items were understandable and none required changes, and the scale was finalized.

### Construct validity

**Figure 1:**
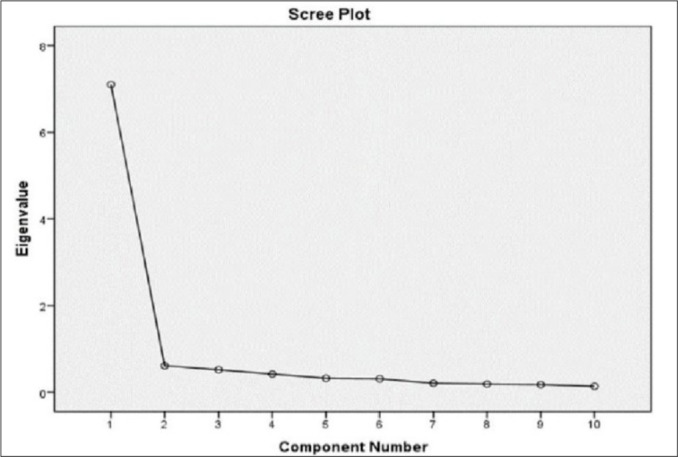
Scree-Plot Graph of the Factor Analysis.

We assessed the suitability of our sample size for factor analysis with the KMO test and Bartlett’s significance test. The KMO coefficient should be above 0.60, and Bartlett’s sphericity test calculated in the chi-square test should be statistically significant^28^. In our study, the KMO coefficient was 0.94, and Bartlett’s significance test was statistically significant (χ^2^=1652.80, df=45, *p*<.001), indicating that our data were suitable for factor analysis. The 10 items were thus analyzed via EFA, using PCA with direct oblimin rotation. Inconsistent with the original scale, PCA revealed only one factor with an eigenvalue above 1. However, as the scree plot revealed two factors, we decided to rerun our PCA analysis and finally identified two factors, as found in the original scale^15^. The scree plot is presented in Figure 1 and the scale’s factor structure is outlined in Table 2.

**Table 2:** Factor structure of Turkish APSQ with principle components analysis.

Items	Factor 1	Factor 2
1. I worry about the amount of sleep I am going to get every night	.629	
2. I worry about how the amount of sleep I had last night is going to affect my day time performance	.983	
3. I worry about how the amount of sleep I get is going to afflict my health	.888	
4. I worry about how much the amount of sleep I get will weaken my social ability	.907	
5. I worry about how much the amount of sleep I get will shake my moo	.661	
6. I worry about my loss of control over sleep		.649
7. I worry about my ability to stay awake and alert during the day	.838	
8. I put great effort into trying to rectify my sleep problems		.566
9. My failure to rectify my sleep problems troubles me a lot		.832
10. My worry about sleep is persistent		.825

**Notes:** Only loadings above 0.3 are presented in the table. Factor 1: Worries about the consequences of poor sleep; Factor 2: Worries about uncontrollability of sleep.

**Figure 2:**
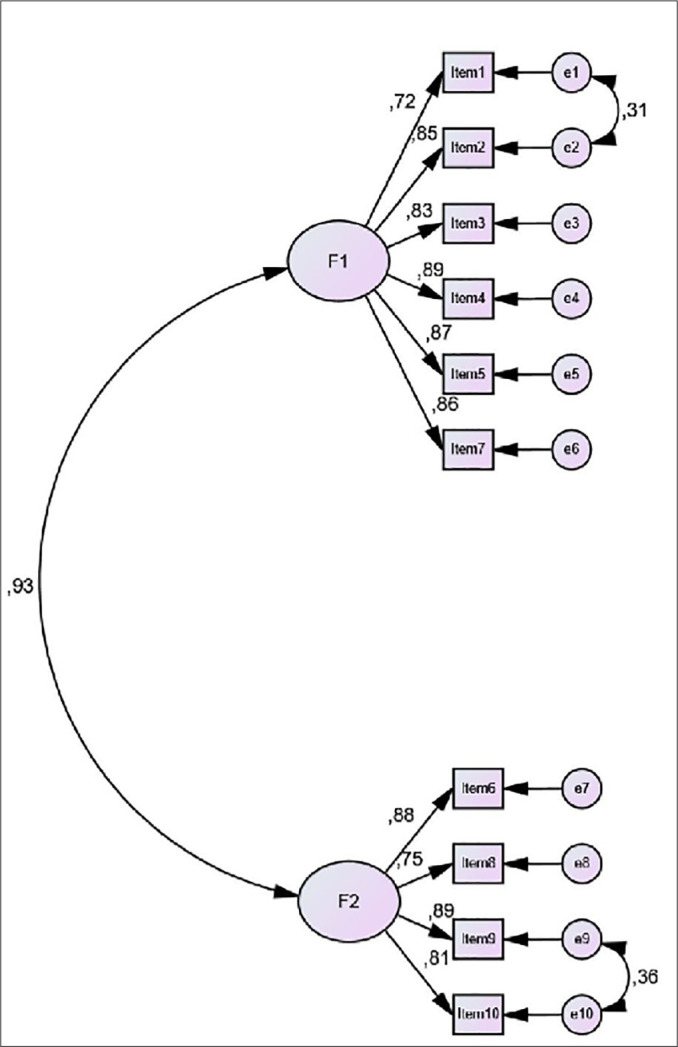
Confirmatory factor analysis Path diagram for two factor model of Turkish version APSQ and standardized factor loadings - F1: Worries about the consequences of poor sleep and F2: worries about uncontrollability of sleep.

In addition to EFA, we performed CFA to further test the two-factor model. The standardized factor loads of scale items were found to vary from 0.72 and 0.89. CFI, GFI, IFI>0.900, χ^2^/df<5, and RMSEA<0.0854 values are recommended as criteria for indicating a good fit indices except if RMSEA were suitable^26,28^. CFA results suggested a modification to improve the goodness of fit indices. After combining the error variances of items 1 and 2 and of items 9 and 10, all fit indices reached acceptable levels. Confirmatory factor analysis path diagram is shown in Figure 2 and the fit indices of the scale are shown in Table 3.

**Table 3:** Model-fit results of confirmatory factor analysis for Turkish version APSQ, primary and after modification.

Model	RMSEA	CFI	GFI	IFI	χ^2^/df	p
Two-factor model with 10 items before the first modification Two-factor model with 10 items 1^st^ modification	.099 .089	.963 .971	.908 .918	.963 .971	2.796 2.434	<0.000 <0.000
Two-factor model with 10 items 2^nd^ modification	.078	.979	.930	.979	2.100	<0.000

**Notes:** RMSEA = Root mean square error of approximation; CFI = Comparative fit index; GFI = Goodness of fit index; IFI = Incremental fit index; χ^2^/df = Normalized chi-square.

### Internal consistency and test-retest reliability

Cronbach’s alpha correlation analysis was used to determine the scale’s internal consistency, and item analysis was used to determine the quality of items. Cronbach’s alpha values of total scale, factor 1 and factor 2 were 0.95, 0.935, and 0.9, respectively, indicating excellent internal consistency. Table 4 lists the item-total score correlations and Cronbach’s alpha values that would result from deleting each item. The test-retest correlation for MDD patients coefficient was calculated as r=0.661 (n=20; *p*<0.001) and for non-clinical population as r=0.828 (n=25; *p*<0.001).

**Table 4:** Item-total statistics for Turkish APSQ in study group.

Item	Mean	Mean	Corrected Item-Total Correlation	Cronbach’s Alpha if item deleted (α)
Item 1	4.18	3.51	.726	.952
Item 2	4.16	3.25	.825	.948
Item 3	4.28	3.30	.801	.949
Item 4	4.48	3.37	.842	.948
Item 5	4.51	3.52	.837	.948
Item 6	3.97	3.38	.833	.948
Item 7	4.36	3.47	.816	.949
Item 8	3.98	3.48	.717	.953
Item 9	3.71	3.43	.850	.947
Item 10	3.53	3.42	.767	.951

APSQ:Anxiety and Preoccupation about Sleep Questionnaire; SD:Standart deviation.

### Criterion and predictive validities

To assess criterion validity, Pearson correlations were computed between Turkish APSQ scores and scores on the PSQI, and the ISI. As it can be observed in the Table 5, criterion validity was demonstrated because high correlations were found between scores of the Turkish APSQ, PSQI, and the ISI.

**Table 5:** Correlations of Turkish APSQ with other measures.

Scale and sub-scale	PSQI	ISI
APSQ	.787*	.749*
Worries about the consequences of poor sleep	.688*	.655*
Worries about uncontrollability of sleep	.756*	.739*

Notes:

* p<.01; APSQ = Anxiety and preoccupation about sleep questionnaire; PSQI = Pittsburg sleep quality index; ISI = Insomnia severity index.

In addition, the predictive validity of the scale was analyzed via independent-samples t-test and we found significantly higher scores among the depression group (M=67.54, SD=24.61) compared to university students (M=33.35 SD=24.92; t=7.826, p<0.0001). According to ISI, while 75% (n=30) of the depression group had clinical insomnia, 17.8% of the control group was detected clinical insomnia. Turkish APSQ scores as other sleep scales were significantly higher in the clinical insomnia group compared to good-sleepers group. Table 6 shows comparisons of sleep scale scores between good-sleepers group and clinical insomnia group.

**Table 6:** Comparisons of sleep scale mean scores between good-sleepers group and clinical insomnia group.

	Good-sleepers	Clinical insomnia	p
Control group n(%)	116(82.2)	25(17.8)	
Depression group n(%)	12(25)	30(75)	
APSQTOTAL	28.69	70.30	<0.001
APSQF1	18.71	40.36	<0.001
APSQF2	9.42	27.07	<0.001
PSQI	5.13	13.67	<0.001
ISI	7.88	19.65	<0.001

**Notes:** APSQ = Anxiety and preoccupation about sleep questionnaire; F1 = Factor 1: worries about the consequences of poor sleep; F2 = Factor 2: worries about uncontrollability of sleep; PSQI = Pittsburg sleep quality index; ISI = Insomnia severity index; n: Number of individuals.

## DISCUSSION

The aim of the current study was to adapt the APSQ to Turkish and examine its psychometric properties in clinical and non-clinical samples. To the best of our knowledge, only two studies have examined the psychometric properties of the APSQ, and they only evaluated the English language form. No clinical samples were assessed in these studies, and the temporal stability of the scale was not analyzed^14,15^. Therefore, this is the first study to investigate the validity and reliability of the scale in a different language, perform test-retest analysis, and assess the scale with a clinically diagnosed group.

First, we examined the factor structure of the scale. The KMO value greater than 0.60 and a significant Bartlett’s test are required^24,25^. Our analyses yielded a KMO coefficient of 0.94 and a statistically significant Bartlett’s test (χ^2^=1652.80, df=45, *p*<.001), indicating that the scale was suitable for factor analysis.

After applying EFA and PCA with direct oblimin rotation, only one factor emerged with an eigenvalue above 1. However, since the scree plot revealed a two-factor structure and the original scale has a two-factor structure, we forced the scale into a two-factor structure with PCA and direct oblimin, achieving alignment with the original scale. This two-factor structure allows standardization across studies using this scale. Factor loading values are coefficients that describes the relationships between items and factors. In general, a loading value at or above 0.60 is considered high, regardless of its positive or negative values^27^. In our analysis, all items included in a factor had factor loadings greater than 0.60, indicating that the scale has a two-factor structure like the original. CFA was used to reanalyze the factor structure obtained from EFA, resulting in suggested modifications to reach acceptable fit indices. After two modifications (the error variances between item 1 - item 2 and item 9 - item 10 were combined) the fit indices according to CFA are as follows: χ^2^/df=2.100, CFI=979, GFI=.930, IFI=979, RMSEA=.078. The scale items’ standardized factor loads ranged from 0.72 to 0.89. Therefore, through EFA and CFA methods, the Turkish APSQ showed good fit indices and appropriate factor loads in our sample.

We also used Cronbach’s alpha to assess the scale’s internal consistency. Cronbach’s alpha coefficients were .952, .935, and .906 for the total scale and two subscales, respectively. These values are similar to those found in previous studies (.93, .91, and .86)^14,15^. As a Cronbach’s alpha coefficient above 0.80 indicates that the scale is highly reliable^28^, the Turkish APSQ has very good internal consistency.

In reliability analysis, item-total score correlation coefficients must be greater than 0.30. Our item analysis results revealed corrected item-total score correlation coefficients between 0.71 and 0.84, high correlations that suggest that the scale items were understood correctly by participants^29^. One important parameter of scale reliability is yielding similar results from the same participant at different times. It is desirable for a scale’s test-retest correlation to be at least 0.70^30^.

Our study is the first to examine the test-retest reliability of the APSQ by readministering the scale to randomly selected samples from both groups (20 MDD patients and 25 controls) two weeks after the initial administration and analyzing results for temporal stability.

The Pearson correlation coefficients were 0.661 for MDD patients and 0.828 for non-clinical sample. Psychiatric interventions such as psychopharmacological treatments and/or sleep hygiene training with the MDD patients may have affected the stability of their scores over these two weeks. This suggests that the scale may be useful in reflecting a course of treatment.

To determine criterion validity, relationships between Turkish APSQ scores and scores from the PSQI, and the ISI were examined. As found in previous studies, high levels of anxiety and preoccupation about sleep were significantly correlated with bad sleep quality, late sleep onset, and insomnia severity^14,15^. Mean APSQ scores were significantly higher in the depression group than in the non-clinical group (67.54 versus 33.35), indicating that sleep-related anxiety was higher in MDD patients. While sleep-related anxiety and preoccupation among MDD patients have not been investigated in previous studies, this result suggests that sleep-related anxiety in depression patients may mediate insomnia^14,15^. Depression and insomnia pose a chicken or egg question. Insomnia can lead to depression but can also be a symptom of depression, and treating insomnia improves the treatment of depression^31^. The APSQ can detect sleep-related anxiety in patients with depression, prompting appropriate psychiatric interventions that will not only treat insomnia but also depression.

### Limitations

Our study has some limitations. First, it includes a relatively small clinical sample and the non-clinical sample included only university students. Therefore, its results cannot be generalized to the general population or all patients with depression. Second, the age and gender distribution were not balanced in both groups. Third, the present study was cross-sectional, precluding the determination of causal relationships. Fourth, correlations between APSQ scores and anxiety and depression levels could not be examined, since scales measuring depression and anxiety were not administered. Future studies should examine the psychometric properties of the APSQ in larger patient groups.

## CONCLUSION

Here, we showed that Turkish APSQ is valid and reliable scale for assessing anxiety and preoccupation about sleep in both clinical and non-clinical populations. We found that anxiety and preoccupation about sleep are associated with impaired sleep quality and severity of insomnia in both MDD patients and non-clinical controls. Thus, measuring anxiety and preoccupation about sleep can guide insomnia treatment or clinical research. It is therefore important to analyze the validity and reliability of the APSQ across a variety of general populations and clinical groups.
